# Low Power Upconversion Mixer for Medical Remote Sensing

**DOI:** 10.1155/2014/923893

**Published:** 2014-07-08

**Authors:** De Xing Lioe, Suhaidi Shafie, Harikrishnan Ramiah, Gim Heng Tan

**Affiliations:** ^1^Department of Electrical and Electronics Engineering, Faculty of Engineering, Universiti Putra Malaysia, 43400 Serdang, Selangor, Malaysia; ^2^Institute of Advanced Technology (ITMA), Universiti Putra Malaysia, 43400 Serdang, Selangor, Malaysia; ^3^Department of Electrical Engineering, Faculty of Engineering, University of Malaya, 50603 Kuala Lumpur, Malaysia; ^4^Department of Electrical and Electronic Engineering, Segi University, 47810 Petaling Jaya, Selangor, Malaysia

## Abstract

This work presents the design of a low power upconversion mixer adapted in medical remote sensing such as wireless endoscopy application. The proposed upconversion mixer operates in ISM band of 433 MHz. With the carrier power of −5 dBm, the proposed mixer has an output inferred 1 dB compression point of −0.5 dBm with a corresponding output third-order intercept point (OIP3) of 7.1 dBm. The design of the upconversion mixer is realized on CMOS 0.13 *μ*m platform, with a current consumption of 594 *μ*A at supply voltage headroom of 1.2 V.

## 1. Introduction

Mixer is the lifeline of the upconversion process in a transmitter, coupled with the integrating voltage controlled oscillator (VCO) and power amplifiers (PA) circuit defining the transmitter. An upconversion mixer is tasked upon performing frequency translation from a baseband frequency to radio frequency (RF). The supply voltage headroom limitation aligned with the scaling of CMOS technology has initiated efforts upon the realization of low power architecture deemed crucial in portable wireless electronic devices in prolonging the battery lifetime. The limitation of voltage headroom and the degradation of the performance at high operating frequency arise concurrently with technology scaling [1].

The highlight of performance indicator for upconversion mixer design is the linearity and power consumption. Passive mixers do provide comparatively superior linearity performance in comparison to an active architecture, with a penalty paid in conversion loss, often requiring a mandatory larger carrier signal power. Large carrier signal power translates to higher power consumption, while low carrier signal power is undesirable for passive mixer integration [2, 3]. Gilbert cell mixer is the default conventional double balanced architecture in radio frequency integrated circuit (RFIC) realization. The advantage of Gilbert cell mixer is in its superior port-to-port isolation and its ability in cancelling off undesired RF and local oscillator (LO) output signal feedthrough in the midst of providing higher conversion gain. In the evolution of technology scaling with a proportional lower supply headroom the fact that Gilbert cell mixers integrate high number of series stacked transistors limits the endeavor in achieving low power consumption, [4–11].

Various architectures had been proposed to combat the constraint of voltage headroom limitation [7–9]. Folded-switching is a promising alternative in operating at low supply voltage abstaining significance effect on the gain and linearity performance [1]. However, with an additional integration of biasing current source, noise is added into the circuit, while the power consumption is ramped up. An alternative solution for low voltage headroom implementation is the switched-transconductance, where the switching stage and transconductance stage are swapped in position respective to the conventional double-balanced mixer [4]. This architecture solves the setback of stacking transistors, while upholding the performances with lower power consumption.

The switching pairs of the transconductance stage are turned on and off alternately. The principle of frequency translation is akin to double-balanced topology. The aspect ratio in the width of PMOS to the width of NMOS is twice in magnitude justifying different carrier mobility between PMOS and NMOS devices. At node 〈*a*〉 of Figure 2, for a positive half cycle of LO signal,* M*_3_ is switched off while* M*_1_ is switched on, resulting in the transconductor to be switched on. The reversed operation is observed at node 〈*b*〉. Push pull configuration of switching stage is adopted instead of single NMOS switch due to larger output swing and thus providing superior noise immunity.

This paper presents the design of low power switched transconductance mixer targeted towards portable medical devices at 433 MHz operating frequency, simulated in 0.13 *μ*m CMOS technology. The operating frequency of 433 MHz is selected in consideration of the in-body wireless communication losses. The range of frequency with least losses is found to be between 400 MHz and 900 MHz [12], which confirms the industrial, scientific, and medical (ISM) band with frequency ranging from 433.05 MHz to 434.79 MHz, and the medical implant communication services (MICS) with frequency of 402–405 MHz. ISM band is chosen as the preferred operating frequency over MICS although the latter is reserved by Federal Communications Commission (FCC) for implantable medical devices as MICS has low data rate up to a permissible level of 500 kbps.

The paper is organized as follows.  Section 2 discusses the architecture and operation of the proposed upconversion mixer. The simulation result is presented in Section 3 in verifying the performance. Finally the conclusion is given in Section 4.

## 2. Mixer Architecture and Operation

**Figure 1:**
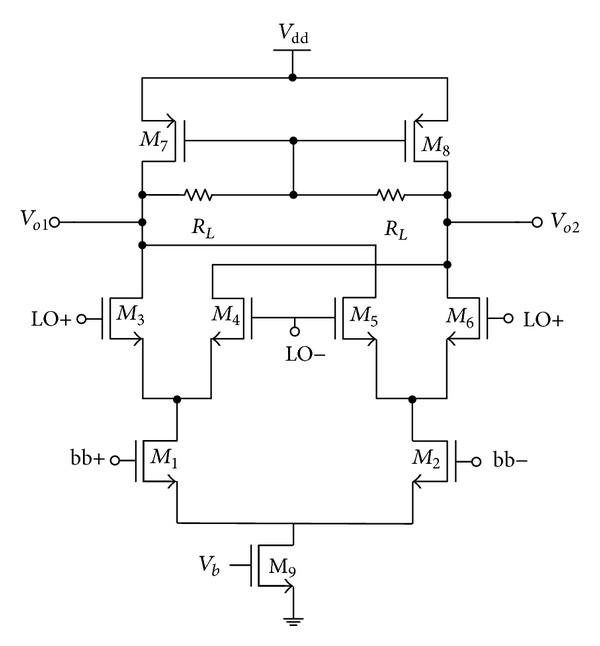
Conventional double-balanced mixer.

Figure 1 shows the architecture of the conventional double-balanced mixer encapsulating the transconductance stage (*M*_1_,* M*_2_), switching stage (*M*_3_–*M*_6_), and the output load resistor, *R*_*L*_.

**Figure 2:**
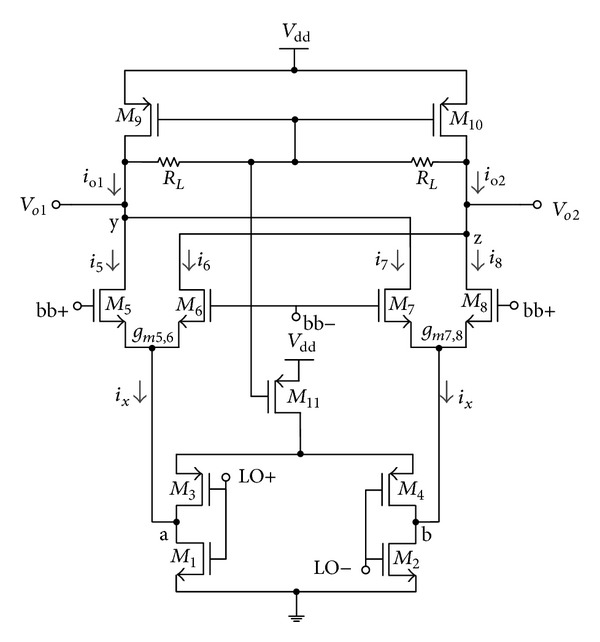
Proposed upconversion mixer.

The transconductance stage converts the input voltage to current signal, which is subjected to mix with LO or carrier signal in the switching stage. A modified structure of the double-balanced mixer in Figure 1 is proposed in Figure 2 which consists of switching pairs (*M*_1_–*M*_4_), transconductance stage (*M*_5_–*M*_8_), and load stage (*M*_9_,* M*_10_ and *R*_*L*_) coupled with the compensating common-mode feedback structure (CMFB).* M*_11_ is the tail transistor integrating as current source. In comparison with the conventional architecture, the inverting switching stage and transconductance stage are swapped in position.

CMFB structure is adapted where the PMOS transistor* M*_9_ and* M*_10_ operates as current source for the differential signals. Common mode current absorbed by the two PMOS load transistor increases the differential gain by

(1) $$\begin{array}{c} A_{v}=-g_{m}\left(R_{L}||r_{o1}||r_{o2}\right) \end{array}$$

and at the same time allowing wider voltage headroom. It is an encouraging alternative in integrating diode-connected PMOS load rather than resistor loading, even in the event that the advantages of CMFB comes with a trade-off of higher noise introduced to the circuit. The rational of this tradeoff is constructed, such that the noise figure is not paramount in upconversion mixer [5], and the significance of low power consumption in the application of portable medical devices is inevitable.

Transistor* M*_11_ provides dc stability for the mixer integrating as a current source for the switching pair. The biasing voltage of* M*_11_ is fed from CMFB regulating the output voltage and eliminating the need of a redundant bias voltage network, thus relaxing the complexity and active chip area consumption.

The comprehensive operation of the proposed mixer as illustrated in Figure 2 is depicted by the following mathematical expressions. Current through transistor* M*_5_ is

(2) $$\begin{array}{c} i_{5}=g_{m_{5,6}}\cdot \frac{1}{2}v_{\text{bb}}\cdot \frac{1}{2}i_{x}=\frac{1}{4}g_{m_{5,6}}v_{\text{bb}}i_{x}, \end{array}$$

where *g*_*m*_5,6__ is the transconductance for NMOS transistor pair* M*_5_ and* M*_6_, *v*_bb_ = *V*_bb_sin*ω*_bb_ *t* is the input baseband signal, and *i*_*x*_ is the current due to instantaneous LO voltage. Similarly, current through transistor* M*_6_ can be expressed as

(3) $$\begin{array}{c} i_{6}=g_{m_{5,6}}\cdot -\frac{1}{2}v_{\text{bb}}\cdot \frac{1}{2}i_{x}=-\frac{1}{4}g_{m_{5,6}}v_{\text{bb}}i_{x}. \end{array}$$

Current at node 〈*y*〉 can then be obtained as

(4) $$\begin{array}{c} i_{o1}=\frac{1}{2}g_{m_{5,6}}v_{\text{bb}}i_{x}. \end{array}$$

Similarly, current at node 〈*z*〉 is expressed as

(5) $$\begin{array}{c} i_{o2}=-\frac{1}{2}g_{m_{7,8}}v_{\text{bb}}i_{x}. \end{array}$$

The differential mixer output current can be derived as

(6) iout=io1−io2=12gm5,6vbbix−(−12gm7,8vbbix)=gmeffVbbsinωbbt·sq[sinωLOt]=gmeff(4π)Vbbsinωbbt[sinωLOt+13sin3ωLOt+⋯],

where sq[sin*ω*_LO_ *t*] is the square wave function of LO signal and *g*_*m*_eff__ is the effective transconductance of the mixer. The differential mixer output signal is eventually obtained as

(7) Vout=2πRLgmeffVbb ×[cos⁡(ωLO−ωbb)t−cos⁡(ωLO+ωbb)t+⋯].

From (7), the conversion gain of the mixer is observed to be

(8) $$\begin{array}{c} \text{CG}=\frac{V_{\text{out}}}{V_{\text{in}}}=\frac{2}{\pi }R_{L}g_{m_{\text{eff}}}. \end{array}$$

Looking at low voltage perspective, the derivation of overdrive voltage for conventional mixer as in Figure 1 is given as follows:

(9) $$\begin{array}{c} V_{\text{dd}}=V_{{\text{ds}}_{1}}+V_{{\text{ds}}_{3}}+V_{{\text{ds}}_{7}}+V_{{\text{ds}}_{9}}+V_{{\text{th}}_{\;\hat{\;}\;7}}, \end{array}$$

where *V*_ds_ is the overdrive voltage and *V*_th_ is the threshold voltage. Similarly, the supply voltage derived for the proposed mixer in Figure 2 can be derived as

(10) $$\begin{array}{c} V_{\text{dd}}=V_{{\text{ds}}_{1}}+V_{{\text{ds}}_{5}}+V_{{\text{ds}}_{9}}+V_{{\text{th}}_{\;\hat{\;}\;1}}. \end{array}$$

Equation (10) shows that the proposed mixer requires one less overdrive voltage of a transistor for the operation, which leads to reduced DC power consumption.

## 3. Simulation Results

The proposed architecture of the upconversion mixer is simulated and verified using Cadence Spectra RF platform. Components parameters are shown in Table 1. The design is implemented in a 0.13 *μ*m standard CMOS technology at supply headroom of 1.2 V. The results verify that the upconversion mixer consumes a total current of 594 *μ*A which results in a dissipation of DC power of 0.71 mW. Low DC power consumption is essentially vital in the application of wireless medical device such as capsule endoscope.

**Figure 3:**
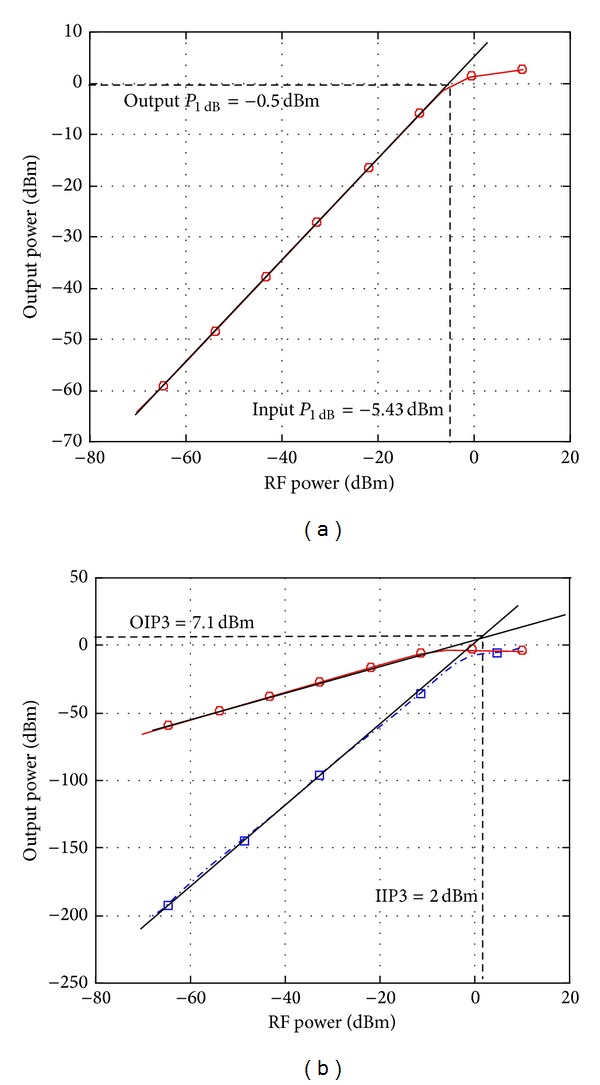
(a) 1 dB compression point. (b) Third-order intercept point.

Figure 3(a) shows the input referred 1 dB compression point of −5.43 dBm and output referred 1 dB compression point of −0.5 dBm. Two-tone analysis is conducted to verify the linearity characteristic as illustrated in Figure 3(b). The input third-order intercept point (IIP3) of 2.0 dBm and output third-order intercept point (OIP3) of 7.1 dBm were achieved with an equivalent gain of 5.4 dB. This agrees with the general relationship of OIP3 = IIP3 + Gain, as also obtained in [1].

**Figure 4:**
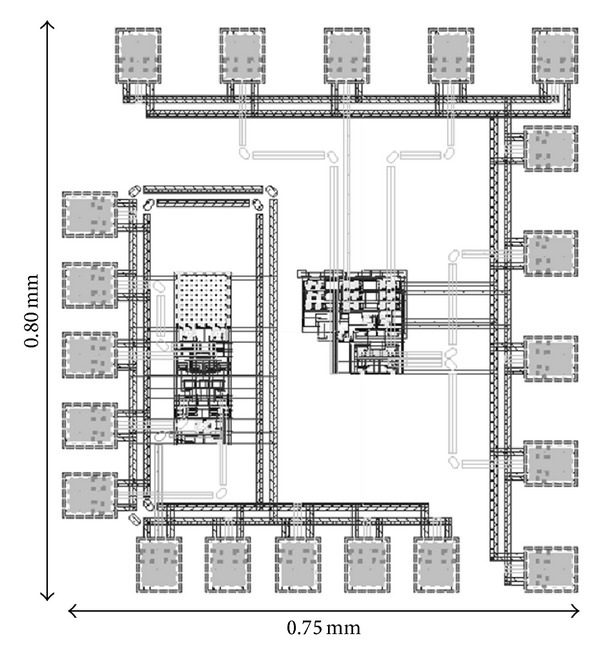
Layout of the proposed mixer.

Table 2 enlists the performance comparison highlighting the proposed mixer respective to recent reported works. From Table 2, the proposed architecture highlights a lead in low power consumption and high linearity performance. Lumping together the performance parameter the comparison is given by a figure of merit (FOM):

(11) $$\begin{array}{c} \text{FOM}=10\log\left[\frac{10^{G/20}\cdot 10^{\text{O}{\text{P}}_{1\;\text{dB}}/20}\cdot \left(\text{Freq}\left(\text{GHz}\right)/1\;\text{GHz}\right)}{\left(P_{\text{DC}}\left(\text{mW}\right)/1\;\text{mW}\right)\cdot \left(10^{P_{\text{LO}}/10}/1\;\text{mW}\right)}\right], \end{array}$$

where *G* is the mixer gain, OP_1 dB_ is the output referred 1 dB compression point, Freq is the operating frequency, *P*_DC_ is the total power consumption with baseband and LO signal, and *P*_LO_ is local signal power in dBm. The FOM originates from the fact that gain and OP_1 dB_ contribute to better performance of a mixer. At the same time, power consumption should be kept as low as possible in measuring a superior performance. Operating frequency is taken into account to obtain a fair comparison with other designs of different operating frequencies. Referring to the FOM comparison in Table 2, the proposed mixer shows a superior overall performance with a FOM of 5.31. The physical layout of the circuit is illustrated in Figure 4.

## 4. Conclusion

This paper presented a low power, high linearity upconversion mixer for the portable medical devices at 433 MHz. The low power architecture integrates regulated bias with inverting switching input. Implemented in 0.13 *μ*m standard CMOS technology the mixer consumes supply headroom of 1.2 V while dissipating 0.71 mW of DC power.

## Figures and Tables

**Table 1:** Simulation parameters used for the proposed mixer of Figure 2.

Components	*W*/*L* (*μ*m)
*M*_1_, *M*_2_	15/0.13
*M*_3_, M_4_	30/0.13
*M*_5_, *M*_6_, *M*_7_, *M*_8_	35/0.13
*M*_9_, *M*_10_	130/0.35
*M*_11_	10/0.35

*R*_*L*_	1.7 kΩ

**Table 2:** Performance comparison.

	[8]*	[9]**	[10]*	[11]**	This work
*V*_DD_ (V)	1.2	1.2	3.0	3.3	1.2
Frequency (GHz)	4.0	23	2.45	0.9	0.434
Gain (dB)	2.3	0.7	12.2	−3.7	5.4
OIP3 (dBm)	5	≈4.8	9	13.3	7.1
OP_1 dB_ (dBm)	—	≈−6.1	1.7	5.3	−0.5
Power (mW)	7.1	8.0	34.2	29.7	0.71
LO power (dBm)	2.0	3.0	8.0	0	−5.0
FOM	—	−1.11	−12.50	−14.39	5.31

*Simulation results. **Measurements results.

## References

[1] Vidojkovic V, van der Tang J, Leeuwenburgh A, van Roermund AHM (2005). A low-voltage folded-switching mixer in 0.18 *μ*m CMOS. *IEEE Journal of Solid-State Circuits*.

[2] Wang X, Andreani P Comparison of the image rejection between the passive and the Gilbert mixer.

[3] Behbahani F, Kishigami Y, Leete J, Abidi AA (2001). CMOS mixers and polyphase filters for large image rejection. *IEEE Journal of Solid-State Circuits*.

[4] Klumperink EAM, Louwsma SM, Wienk GJM, Nauta B (2004). A CMOS switched transconductor mixer. *IEEE Journal of Solid-State Circuits*.

[5] Grau G, Langmann U, Winkler W, Knoll D, Osten J, Pressel K (2000). A current-folded up-conversion mixer and VCO with center-tapped inductor in a SiGe-HBT technology for 5-GHz wireless LAN applications. *IEEE Journal of Solid-State Circuits*.

[6] Cortes FP, Bampi S A 1.4GHz upconversion mixer design using the gm/ID method suitable for a multi-band analog interface.

[7] Tan GH, Sidek RM, Ramiah H, Chong WK (2012). Design of ultra-low voltage 0.5V CMOS current bleeding mixer. *IEICE Electronics Express*.

[8] Murad SAZ, Pokharel RK, Kanaya H, Yoshida K A 3.0–5.0 GHz high linearity and low power CMOS up-conversion mixer for UWB applications.

[9] Verma A, Kenneth KO, Lin J (2006). A low-power up-conversion CMOS mixer for 22–29-GHz ultra-wideband applications. *IEEE Transactions on Microwave Theory and Techniques*.

[10] Choi JY, Lee SG (2004). A 2.45 GHz CMOS up-conversion mixer design utilizing the current-reuse bleeding technique. *International Journal of Electronics*.

[11] Choi K, Yoo S, Kim M (2010). CMOS DSB transmitter with low TX noise for UHF RFID reader system-on-chip. *IEEE Transactions on Microwave Theory and Techniques*.

[12] Kim K, Yun S, Lee S, Nam S, Yoon YJ, Cheon C (2012). A design of a high-speed and high-efficiency capsule endoscopy system. *IEEE Transactions on Biomedical Engineering*.

